# Relationships between changes in pain severity and other patient-reported outcomes: an analysis in patients with posttraumatic peripheral neuropathic pain

**DOI:** 10.1186/1477-7525-9-17

**Published:** 2011-03-25

**Authors:** Robert van Seventer, Michael Serpell, Flemming W Bach, Bart Morlion, Gergana Zlateva, Andrew G Bushmakin, Joseph C Cappelleri, Meryem Nimour

**Affiliations:** 1 Pain Clinic, Amphia Hospital, Breda, The Netherlands; 2 Pain Clinic, Gartnavel General Hospital, Glasgow, UK; 3 Danish Pain Research Center, Aarhus, University Hospital, Aarhus Denmark; 4 Department of Neurology, Hospital of Aalborg, Aarhus University Hospital, Aalborg, Denmark; 5 The Leuven Centre for Algology and Pain Management, University Hospitals Leuven, Belgium; 6 Pfizer Inc., New York, NY, USA; 7 Pfizer Inc., Global Research & Development, New London, CT, USA; 8 Pfizer Limited, Walton Oaks, Tadworth, Surrey, England, UK

## Abstract

**Background:**

The objective of this study is to use the pain numeric rating scale (NRS) to evaluate associations between change in pain severity and changes in sleep, function, and mood assessed via patient-reported outcomes (PROs) in patients with posttraumatic pain.

**Methods:**

This is a secondary analysis of a clinical trial evaluating pregabalin in patients with posttraumatic peripheral neuropathic pain (N = 254). Regression models were used to determine associations between changes in pain (0-10 NRS) as the predictor and scores on the following PRO measures as the outcome: Pain Interference Index; Hospital Anxiety and Depression Scale anxiety and depression subscales; Medical Outcomes Study-Sleep Scale 9-item Sleep Problems Index and Sleep Disturbance subscale; and Daily Sleep Interference Scale (0-10 NRS).

**Results:**

Change in pain severity showed clear, direct relationships with changes in function, anxiety, depression, and sleep PROs, all of which were statistically significant (*P *<.001). Results from subgroup analyses (≥30% or ≥50% pain responders, pregabalin or placebo treatment, age ≤ 51 years or > 51 years) tended to be consistent with results from the overall sample.

**Conclusions:**

Overall, a direct relationship exists between pain and various aspects of patient's well-being and functioning, which can provide a quantitative assessment of how improvements in pain may be expected to relate to other patient outcomes. (http://ClinicalTrials.gov Identifier number NCT00292188; EudraCT #2005-003048-78).

## Background

Because the complexity and subjective nature of pain complicates evaluation of its severity and impact, various patient self-report instruments have been developed to assess pain and other patient-reported outcomes (PROs) in the research and clinical settings [1]. The 11-point numeric rating scale (NRS), which ranges from 0 (no pain) to 10 (worst possible pain), has become one of the most frequently used instruments for evaluating pain based on its simplicity and ease of comprehension by patients. This NRS is recommended by the Initiative on Methods, Measurement, and Pain Assessment in Clinical Trials (IMMPACT) as one of the core outcomes for assessment in clinical trials of chronic pain [2]. Additionally, IMMPACT recommends that function and mood should be included as core outcomes. The presence and increased severity of pain often results in reduced function and increased mood disturbance [3,4]. Although not included in the IMMPACT recommendations, sleep is another outcome that is affected adversely by pain, with consistent evidence endorsing this relationship [5-7].

When evaluating these outcomes, we believe that because the presence of pain generally interferes with daily functions, improvement in pain will be associated with improved functioning and other health benefits such as sleep and mood--specific outcomes that can be quantified and expressed. Therefore, characterizing and quantifying the relationship between pain severity and corresponding levels of interference with daily function, sleep, and mood can inform treatment decisions and guide assessment of outcomes. Previous studies in painful diabetic peripheral neuropathy have evaluated the relationship between pain and other PROs to categorize patients with mild, moderate, and severe pain [8,9]. One of these studies suggested that changes across severity categories correlate with specific score changes in PROs [9]. The purpose of the current study is to use the pain NRS to characterize and quantify in a clinical and meaningful way the extent of the relationship between pain severity scores and scores on other PROs that measure sleep, pain interference on daily functions, and mood.

## Methods

This study is a secondary analysis using data derived from a placebo-controlled clinical trial evaluating the efficacy of pregabalin in patients with posttraumatic peripheral neuropathic pain (N = 254). The methodology and primary analysis of the trial have been reported elsewhere [10].

All patients gave written, informed consent. Institutional review boards reviewed and approved the protocol and the study was conducted in accordance with the Declaration of Helsinki, Good Clinical Practice guidelines and local laws and regulations. Patients were eligible for participation if they received a diagnosis of posttraumatic peripheral neuropathic pain (including post-surgical neuropathic pain, neuropathic pain due to peripheral nerve injury, and phantom limb pain) that was confirmed by a qualified pain specialist and persisted for a minimum of three months following the traumatic event. Patients were enrolled if they had a score of ≥40 mm on the visual analog scale of the short-form McGill Pain Questionnaire and completed ≥ 4 daily pain diaries during the last week of the screening period prior to randomization, with the mean score being ≥4 on the 11-point (0-10) NRS.

Patients with neuropathic pain that was not due to trauma (e.g. diabetic peripheral neuropathy, postherpetic neuralgia, radiculopathy, trigeminal neuralgia or carpal tunnel syndrome), was central rather than peripheral (e.g. spinal cord injury) or was due to Complex Regional Pain Syndrome (Type 1 or Type II) were excluded. Also, patients suffering from clinically significant or unstable conditions that, in the opinion of the investigators, would compromise participation in the study were excluded.

The current report focuses on the association between changes in pain severity and changes in PROs of pain interference on daily functions, sleep, and mood; these analyses are independent of the treatment allocation (pregabalin or placebo) and comparative results reported in the primary analysis.

We evaluated the association between change in pain, assessed daily using a 0-to-10 NRS (0 = no pain, 10 = worst possible pain) and then averaged to give a weekly result, and several other PROs assessed at baseline and end of double-blind treatment at week 8. These PROs included the following: the Pain Interference Index (PII) from the modified Brief Pain Inventory - short form (mBPI-sf) [11], a composite score of the 7 interference items assessed using a 0-to-10 NRS anchored at 0 = does not interfere and 10 = completely interferes (recall period of the past 24 hours); the anxiety and depression subscales of the Hospital Anxiety and Depression Scale (HADS) [12], with each subscale consisting of 7 items scored using a 4-point Likert-type scale (1-week recall period) and higher scores indicating greater severity; the Medical Outcomes Study-Sleep Scale (MOS-SS) 9-item Sleep Problems Index and 4-item Sleep Disturbance subscale [13], both based on a 1-week recall period with higher scores indicating greater sleep problems; and the Daily Sleep Interference Scale that uses an 11-point NRS to describe how pain has interfered with sleep during the past 24 hours (0 = no interference, 10 = completely interferes). Linear models were applied to evaluate the relationship between the change in each of these PROs as the outcome and the change in pain used as a continuous predictor.

The above-specified relationships between change in pain and PROs were examined using linear regression models. The changes in PRO scores were evaluated as a function of the change in pain NRS score (from baseline to end point).

The model was populated with all available patients who provided data in the clinical trial regardless of treatment allocation or treatment effects. To evaluate the model for consistency and robustness, six sensitivity analyses were performed using subgroups from the clinical trial. These cohorts included patients achieving ≥30% pain response (30% responders), patients achieving ≥50% pain response (50% responders), pregabalin-treated patients, placebo-treated patients, patients aged ≤ 51 years and patients > 51 years. Fifty-one years was chosen as the cut-off value since it is the median age of all patients. The 30% and 50% responders are those patients who achieved at least a 30% and 50% reduction in pain NRS scores, respectively, from baseline to endpoint.

All analyses were performed using SAS version 9.2 (SAS Institute Inc., Cary, North Carolina, USA). A *P *value <.05 was taken to confer statistical significance.

## Results

The population consisted of 254 patients with a mean age of 51.7 years; 50.8% of the patients were female. In the original placebo-controlled clinical study [10], pregabalin was associated with a statistically significant improvement in pain compared to placebo, and significant improvements in other PRO scores that included pain-related sleep interference, the MOS sleep scale (overall sleep problems index, as well as the sleep disturbance and sleep adequacy subscales), and the anxiety and depression subscales of the HADS.

**Figure 1:**
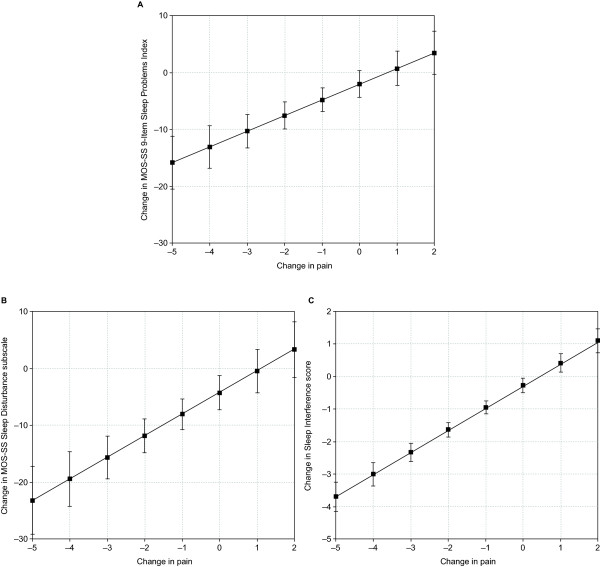
**Predicted relationship between change in pain severity and mean change in severity of patient-reported sleep disruption**. Relationship between change in pain severity and change in severity of patient-reported sleep disruption based on the Medical Outcomes Study-Sleep Scale (MOS-SS) 9-item Sleep Problems Index (A), MOS-SS Sleep Disturbance subscale (B), and sleep interference on a 0-to-10 numeric rating scale (C). Analysis based on the total sample of all available patients. *P *<.001 for the overall relationship.

**Figure 2:**
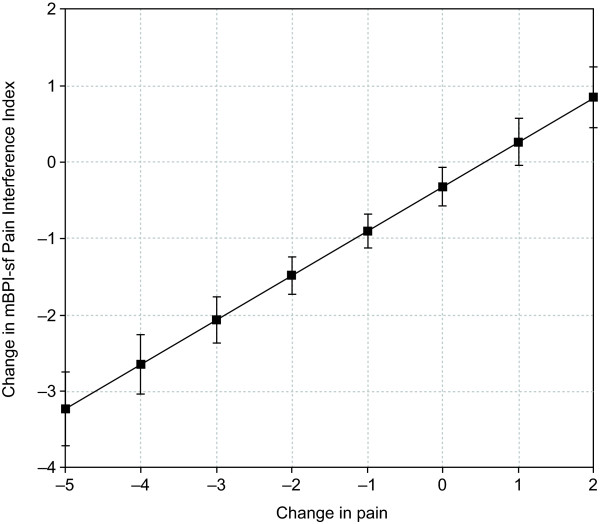
**Predicted relationship between change in pain severity and mean change in pain interference on daily function**. Relationship between change in pain severity and change in pain interference on daily function assessed using the modified Brief Pain Inventory-short form (mBPI-sf) Pain Interference Index. Analysis based on the total sample of all available patients. *P *<.001 for the overall relationship.

**Figure 3:**
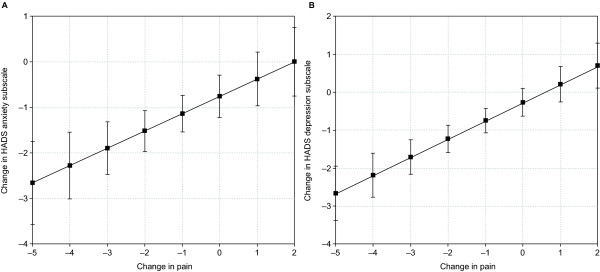
**Predicted relationship between change in pain severity and mean changes in anxiety and depression**. Relationship between change in pain severity and mood based on the anxiety (A) and depression (B) subscales of the Hospital Anxiety and Depression Scale (HADS). Analysis based on the total sample of all available patients. *P *<.001 for the overall relationship

In this secondary analysis, changes in PRO scores were evaluated as a function of change in pain severity. Regression models resulted in linear plots (see Table 1 for slope and intercept estimates) that showed significant associations (*P *<.001) between changes in pain and changes in patient-reported sleep disruption (Figure 1), pain interference on daily functions (Figure 2), and mood (anxiety and depression; Figure 3). For example, a 2-point decrease (improvement) in pain corresponded to an estimated 7.6-point decrease (improvement) in the MOS Sleep Problem Index 9, 11.9-point decrease in MOS Sleep Disturbance, and 1.6-point decrease in Sleep Interference (Figure 1). A 2-point decrease in pain was associated with an estimated 1.5-point decrease in pain interference on daily function or PII (Figure 2). A 2-point decrease in pain was associated with an estimated 1.5-point decrease in HADS anxiety and a 1.2-point decrease in HADS depression (Figure 3). The derived plots can be interpreted as showing, at the individual patient level, the mean change in PRO score (y-axis) that can be expected with the various incremental changes in pain severity (x-axis).

**Table 1:** Slope and intercept estimates from models predicting relationships between changes in pain severity and PROs for all patients

	ESTIMATE (95% CI)					
	MOS-SS DISTURBANCE	MOS-SS 9-ITEM SLEEP PROBLEMS INDEX	SLEEP INTERFERENCE (NRS)	HADS ANXIETY	HADS DEPRESSION	PII

Intercept	-4.30 (-7.31, -1.28)	-2.07 (-4.42, 0.27)	-0.27 (-0.49, -0.05)	-0.76 (-1.22, -0.30)	-0.27 (-0.63, 0.08)	-0.32 (-0.56, -0.08)
						
Slope	3.79 (2.44, 5.13)	2.76 (1.71, 3.80)	0.68 (0.59, 0.78)	0.38 (0.17, 0.59)	0.48 (0.32, 0.64)	0.58 (0.47, 0.69)

Table 2 presents the mean improvement (decrease) in PROs corresponding to a 2-point improvement (decrease) in pain for the total sample. The mean improvement values were estimated as intercept + slope*(-2). For example, using data from Table 1, mean improvement in MOS-SS disturbance that corresponded to a 2-point improvement in pain is -4.3 + 3.79*(-2), which is equal to -11.87, taking into account rounding errors in intercept and slope (Table 2). Table 2 also presents the results of the subgroup sensitivity analyses for 30% and 50% pain responders as well as for individuals in the pregabalin and placebo groups, and individuals aged ≤ 51 years and > 51 years. In general, these sensitivity analyses tended to support the results of the main analysis of the total sample, with some exceptions, for example, 50% responders on the PII scale, and placebo-treated patients as well as patients ≤ 51 years of age on the HADS depression subscale, the MOS-SS Disturbance subscale and the MOS-SS 9-item Sleep Problems Index.

**Table 2:** Mean Improvement in PROs Corresponding to a 2-Point Improvement in Pain for Patients and Preselected Subgroups

	MEAN (95% CI) IMPROVEMENT IN PRO THAT CORRESPONDED TO A 2-POINT IMPROVEMENT IN PAIN					
GROUP	MOS-SS DISTURBANCE	MOS-SS 9-ITEM SLEEP PROBLEMS INDEX	SLEEP INTERFERENCE (NRS)	HADS ANXIETY	HADS DEPRESSION	PII

All patients (N = 254)	-11.87 (-14.83, -8.91)	-7.59 (-9.91, -5.27)	-1.64 (-1.86, -1.43)	-1.52 (-1.97, -1.07)	-1.23 (-1.58,-0.88)	-1.48 (-1.72, -1.25)
Subgroups						
30% responders (n = 82)*	-11.57 (-19.17, -3.98)	-7.41 (-13.31, -1.50)	-1.54 (-2.05, -1.03)	-1.57 (-2.61, -0.53)	-1.39 (-2.13,-0.65)	-1.68 (-2.22, -1.14)
50% responders (n = 48)*	-13.12 (-25.04, -1.19)	-8.37 (-18.07,1.32)	-1.70 (-2.70,-0.70)	-2.04 (-3.88, -0.21)	-1.25 (-2.50,0)	-2.21 (-3.13,-1.28)
Pregabalin- treated patients (n = 127)	-15.79 (-19.76, -11.83)	-9.85 (-13.14, -6.57)	-1.67 (-1.96, -1.38)	-1.64 (-2.25,-1.03)	-1.56 (-2.00,-1.12)	-1.71 (-2.05,-1.38)
Placebo-treated patients (n = 127)	-7.09 (-11.51,-2.68)	-4.93 (-8.22, -1.65)	-1.62 (-1.95, -1.29)	-1.41 (-2.09, -0.74)	-0.83 (-1.39,-0.27)	-1.18 (-1.51,-0.85)
Age ≤ 51 y (n = 127)	-8.12 (-12.35, -3.89)	-5.16 (-8.41, -1.91)	-1.59 (-1.89, -1.30)	-1.17 (-1.83, -0.51)	-0.75 (-1.28, -0.22)	-1.47 (-1.79, -1.15)
Age > 51 y (n = 124)	-15.64 (-19.74, -11.54)	-10.17 (-13.48, -6.86)	-1.69 (-2.01, -1.38)	-1.87 (-2.48, -1.27)	-1.71 (-2.16, -1.26)	-1.50 (-1.85, -1.15)

Mean Improvement = decrease; CI, confidence interval; HADS, Hospital Anxiety and Depression Scale; MOS-SS, Medical Outcomes Study-Sleep Scale; NRS, numeric rating scale; PII, Pain Interference Index; PROs, patient-reported outcomes.

*30% and 50% responders are those patients who achieved at least a 30% and 50% reduction in pain, respectively, in the clinical trial on which this analysis was based.

## Discussion

It is well-recognized that pain affects patient function and may have a reciprocal relationship with specific outcomes such as sleep and mood [3-7]. The results of this study expand our knowledge of the relationship between pain and PRO by suggesting that a clear, direct relationship exists between change in pain and change in patients' self-report of daily function, sleep, and mood. To our knowledge, this is the first study that provides evidence for such a relationship. This study demonstrates a direct quantitative linkage of these relationships, whereby specific changes in pain severity can be mapped to the specific magnitude of a change in a PRO.

Pain severity is not necessarily linearly related to functional impairment [14]. Nevertheless, our imposition of linearity on the relationship between change in pain and change in functional impairment is useful because it enables us to predict and quantify the functional improvement that may be expected to result from successful analgesic treatments. The strength of our results is that they are hypothesis- and empirically-driven findings that are independent of treatment allocation. This model for evaluating change in pain severity can be interpreted at the individual patient level over time, and can be used to determine the expected change in PRO score that corresponds to a particular incremental change in pain severity for a particular patient in the current study from baseline to week 8. This relationship can be explained to the patient and can help to convey the level of improvements that may be achievable, thereby enabling the patients to form more realistic and objective goals.

Even though there were fewer patients in the sensitivity analyses, the results were generally consistent with the main analysis, indicating the invariance of the observed relationships. The few exceptions may be owing to the smaller number of observations and the curtailment of distribution of responses, which resulted in a narrower range in change scores in the subgroups. While the reason for these exceptions warrants further investigation, the overall comparability of the values among the evaluated samples suggests the model's robustness.

Of particular interest is the observation that for some of the PROs, there was a change in score even with no change in pain severity. For example, individuals with no change in pain severity still showed a 4.3-point improvement on the MOS-SS Sleep Disturbance subscale. This can be taken to suggest that there may exist effects of treatment that are independent of the effects on pain, which in this case, can be specific effects on sleep.

Further support for this comes from the sensitivity analyses, for which pregabalin-treated patients with no change in pain improved by 7.50 points on the MOS-SS Sleep Disturbance subscale, whereas placebo-treated patients with no pain change improved only by 1.35 points. Direct effects of pregabalin on sleep improvement have previously been suggested using mediation analysis in a study of patients with fibromyalgia [15]. The relationship between pain and sleep is considered to be bidirectional [6,16], and while studies have suggested that sleep disruption may enhance the pain experience [17-19], it is not clear whether sleep improvement in itself can directly improve pain scores or if the improved sleep increases the patient's ability to cope with the pain.

The data in the present study are consistent with results from a recent study by Hoffman et al. [9]. Using data from a randomized, placebo-controlled trial of pregabalin in patients with painful diabetic peripheral neuropathy, a different population from ours, researchers derived pain severity cutpoint categories on a 0-10 point pain NRS, and compared the magnitude of within-patient change in pain severity with corresponding changes in function and health status. The cutpoint analysis indicated that pain severity ratings of 1-3, 4-6, and 7-10 corresponded to mild, moderate and severe pain, respectively. For each change category, mean (± standard deviation, SD) score changes were examined for the mBPI-sf PII and the Euro-Qol (EQ-5D). On the mBPI-sf PII (0-10 NRS), mean changes of -5.5 (± 2.1) corresponded to a shift from severe pain to no/mild pain; -3.3 (± 2.1), severe to moderate; -3.2 (± 2.1), moderate to no/mild; -0.9 (± 2.0), no change; and 0.4 (± 2.6), worsening (*P *< .0001). Mean changes in the PII ranged from -4.5 (± 2.2) for patients with ≥ 50% NRS reduction and -0.2 (± 2.0) for patients with < 10% NRS reduction (*P *< .0001). Similar differences were observed for the EQ-5D. Thus, changes in pain severity were associated with changes in daily functioning and health status, findings similar to those reported in the presented study.

A two-point reduction on pain is taken to correspond to a clinically meaningful improvement on the other PROs. Psychometric studies on specific PRO scales provide evidence supporting the clinical importance of these changes. For example, a large study assessing the psychometric properties of the Daily Sleep Interference Scale demonstrated significant correlations between this scale and other outcome measures, including pain, and the results suggest that a 1-2 point change from baseline to end of treatment may be interpreted as clinically important [20], a change consistent with values reported in the present study.

In a similar study that evaluated the reliability and validity of the MOS Sleep Scale in patients with painful diabetic neuropathy [21], the MOS Sleep Problems Index was shown to be responsive to clinical changes, with improvements being greater as the pain and sleep of patients improved. Minimal improvement in health status (on measures of pain, sleep, patient or clinical global impression of change) corresponded to mean changes on the MOS Sleep Problems Index that ranged from -10 to -14, a range that overlaps with the mean ± 95% CI reported herein, particularly in pregabalin-treated patients.

A study evaluating the psychometric properties of the BPI for painful diabetic peripheral neuropathy showed that scores on the PII (subscale of BPI) correlate highly and significantly with other outcome measures related to pain, sleep, health status, quality of life and mood [11]. Furthermore, in patients with painful diabetic neuropathy treated with pregabalin, a one-grade reduction in pain level, either "severe-to-moderate" or "moderate-to-no/mild" corresponded to mean (± SD) reductions in the PII of -3.3 (± 2.1) and -3.2 (± 2.1), respectively [9]. The PII values reported herein for all patients and subgroups are below these values but within the mean ± SD range, and therefore may be associated with clinically meaningful changes in pain levels. According to IMMPACT recommendations, a 1-point reduction in the PII may reflect minimally important improvement [22].

An important limitation of this study is that while it may be reasonable to expect that reductions in pain severity will result in improvements in other outcomes, the associations demonstrated by the reported data do not imply causation. Furthermore, these data are derived from patients with nonmalignant chronic pain and do not imply a general physiologic or pathophysiologic parallel regulation. For example, it has been shown that change in depression scores is not necessarily paralleled by change in pain thresholds in patients treated for major depression [23]. The use of other analytic techniques, such as path or mediation analysis, may help further characterize the causal relationship of these associations.

The generalizability of this study is another limitation that should be considered when interpreting the results within the context of clinical trials or clinical practice. Consequently, these results should be used as a guide for further exploring the relationships underlying pain and pain interference with function.

Models with pain as a categorical predictor, which do not impose any functional relationship between outcome and predictor, were also investigated. Results of these models (not reported here) supported the results with pain as a continuous predictor. We believe that the best choice to depict the appropriateness of a model is through probability plots and residual plots. If the probability plot forms a linear pattern and the residual plot forms no pattern, then we can conclude that the fitted relationship (in this case linear) is appropriate. We extensively studied these two types of plots and found the model to be suitable. The use of pain as a continuous predictor not only increases the sensitivity of observed relationships but also lends a simplified and meaningful interpretation of the relationship through the slope as a measure of change.

## Conclusions

In summary, the results reported here provide evidence of a direct and tangible relationship between pain and PROs in patients with chronic, nonmalignant neuropathic pain. Importantly, the data additionally demonstrate that pain responders show other benefits that are quantifiable in relation to the change in pain severity and are clinically significant. This novel analysis can be applied for determining individual responses that can be expected in patients being treated for pain, with the observed relationships providing a framework for quantitatively assessing how improvements in pain may be expected to result in improvement in other patient-centered outcomes. Such information may be useful in the research setting for trial design and in the clinical setting for informing treatment decisions and enhancing assessment of outcomes. Additional confirmatory studies are encouraged.

## Competing interests

RV, MS, FWB, and BM were investigators in this study but were not compensated for the development of this manuscript.

In the past 5 years, RV has received a fee from Pfizer for speaking at an international conference, and his institution received financial compensation for conducting this study.

MS is a consultant and senior lecturer, and works for the Greater Glasgow & Clyde NHS Trust and for the University of Glasgow. In the past 5 years, he has been a member of an advisory board for NAPP and Astellas. He has received honoraria and expenses for lectures or chairing educational meetings from NAPP, Pfizer, Grünenthal and GW Pharmaceuticals. His institution has also received remuneration for performing clinical drug trials from NAPP, Pfizer, Grünenthal, GW Pharmaceuticals, CeNeS and GlaxoSmithKline.

FWB has received honoraria for teaching sessions and advisory board services from Pfizer, MSD, Norpharma, and Eli-Lilly.

BM is a speaker, consultant, and/or clinical science investigator for several pharmaceutical companies involved in analgesics research, but receives no royalty (cash or otherwise) from the sale of any product.

This study was funded by Pfizer. GZ, AGB, JCC, and MN are full-time employees of Pfizer. GZ and AGB are Pfizer stockholders. Pfizer provided funding for journal charges.

## Authors' contributions

The authors prepared this manuscript in collaboration. Each author made substantial contributions to the study conception and design, acquisition of data, statistical analysis, drafting of the manuscript, and final approval of the manuscript.
